# Social cognition in neuropsychiatric populations: a comparison of theory of mind in schizophrenia and mesial temporal lobe epilepsy

**DOI:** 10.1038/s41598-017-00565-2

**Published:** 2017-03-28

**Authors:** Łukasz Okruszek, Aleksandra Bala, Małgorzata Wordecha, Michał Jarkiewicz, Adam Wysokiński, Ewa Szczepocka, Aleksandra Piejka, Oliwia Zaborowska, Marta Szantroch, Andrzej Rysz, Andrzej Marchel

**Affiliations:** 1 Clinical Neuroscience Lab, Institute of Psychology, Polish Academy of Science, Warsaw, Poland; 2 Faculty of Psychology, University of Warsaw, Warsaw, Poland; 3 Third Department of Psychiatry, Institute of Psychiatry and Neurology, Warsaw, Poland; 4 Department of Old Age Psychiatry and Psychotic Disorders, Medical University of Łódź, Łódź, Poland; 5 Department of Neurosurgery, Medical University of Warsaw, Warsaw, Poland

## Abstract

Social cognition deficits are observed both in patients with schizophrenia (SCZ) and in patients with mesial temporal lobe epilepsy (MTLE). This may be due to dysfunction of the amygdala network, which is a common feature of both diseases. In this study, SCZ (n = 48) or MTLE (n = 31) and healthy controls (HC, n = 47) completed assessments of mentalising (Reading Mind in the Eyes Test, RMET) and basic cognitive processing, e.g., working memory, executive functions and psychomotor speed (Trail-Making Test B and Digit Symbol). SCZ were also assessed with the Positive And Negative Syndrome Scale (PANSS). We found that the RMET scores of the two clinical groups were similar (*p* > 0.05) and lower than in the HCs (SCZ: *p* < 0.05; MTLE: *p* < 0.001). In the next step, SCZ were split into two groups with respect to the level of symptoms. Analysis of the RMET scores revealed no differences between the HC (*M* = 25.7 ± 4.1) and POS-LO (*M* = 25.3 ± 4.8); both groups outperformed the POS-HI group (*M* = 21.3 ± 5.2) and the MTLE group (*M* = 20.8 ± 4.6). No differences were found for the median-split with regard to negative symptoms. In SCZ, the mind-reading deficit appears to be associated with the level of positive symptoms. Both POS-HI and MTLE patients present significant mentalising deficits compared to healthy controls.

## Introduction

During the last two decades, “Theory of Mind” (ToM) and its “mentalising/mind reading” processes, understood as the ability to attribute mental states of others^1, 2^, have become some of the most studied areas in cognitive neuroscience^1, 2^. Moreover, the observation that deficient mentalising, alongside other more basic social cognitive deficits, contributes significantly to functional impairment and lower quality of life in patients with neuropsychiatric conditions^3^, making it one of the primary focuses of research in clinical neuroscience as well^4^.

Interdisciplinary cross-fertilisation has been cited as one of the most important drivers of the development of social neuroscience^5^. The relationship between social and clinical neuroscience is reciprocal, with observations of social cognitive deficits in various disorders informing basic social neuroscience research on the one hand and the results of social neuroscience research being used to improve the patients’ treatment on the other^6, 7^.

A recent meta-analysis concluded that the medial prefrontal cortex (mPFC) and bilateral posterior temporo-parietal junction (TPJ) are “core regions” that are activated whenever a situation requires mentalising skills^2^. A number of cortical structures, including the TPJ, the mPFC, the precuneus, the temporal lobes and the inferior frontal gyri, have also been linked to specific mind-reading tasks^2^.

The Reading Mind in the Eyes Test (RMET)^8^ is one of the most extensively used tasks in social neuroscience research. During the task, the participant is presented with an image of a pair of eyes and four single-word descriptors. The respondent has to choose the word that best represents the state of the person whose eyes are shown in the image. The options relate to the emotions and intentions of the target person, so it is presumed that the RMET measures mind reading abilities^9^. A review of neuroimaging studies concluded that the “core ToM network” (mPFC, TPJ) plus the bilateral inferior frontal gyrus (BA 45) are the areas most consistently activated during performance of the RMET^2^. However, unlike other mentalising tasks based on verbal vignettes (e.g., Faux Pas task^10^, False Belief task^11^) or abstract shapes (e.g., Moving Shapes task^12^), the RMET requires participants to use basic social cognitive abilities, associated with gaze processing, in order to make inferences about complex mental states. This has led researchers to emphasise^9^ that the RMET is a test of both emotion processing^13^ and mentalising abilities^14^. Successful RMET task performance is highly dependent on emotion perception and recognition skills. Although it is not a consistent finding, a significant proportion of functional magnetic resonance imaging (fMRI) studies of the neural correlates of RMET have reported activation of subcortical structures, particularly the amygdala, during task performance^15–18^. This evidence of subcortical involvement is corroborated by lesion studies. A study comparing patients with amygdala damage, patients with non-amygdala brain damage and healthy controls found that the group with amygdala damage showed impairments in the recognition of complex states but not basic emotions when compared with controls and patients with other localised brain damage^13^. Some researchers^19^ have found that patients with unilateral amygdala lesions showed impaired RMET performance when compared with healthy controls, whilst patients with temporal lesions that did not include amygdala regions were unimpaired. Interestingly, no such effects have been observed on other ToM tasks. For example, a recent study found that amygdala lesions do not compromise false-belief reasoning^20^. The medial temporal lobe is directly linked to the amygdala and other temporal structures^21^, and a recent meta-analysis of patients with mesial temporal lobe epilepsy (MTLE) concluded that this group has deficits in both recognition of facial emotions and mind-reading^22^.

Abnormal activity in the amygdala and medial temporal lobe structures has also been reported in schizophrenia^23^, and meta-analyses of neural responses to face stimuli have consistently concluded that people with schizophrenia show abnormal amygdalar responses^24^. Similarly, RMET performance has been found to differ strongly between patients with schizophrenia and healthy controls (*d* = 0.90)^25^. The mind reading deficits observed in patients with schizophrenia are of a similar magnitude to those observed in patients with autism spectrum disorders (ASD), the group for which the task was originally designed^26^.

Although there have been some direct comparisons of mentalising functioning in patients with schizophrenia and patients with other neurological impairments, so far, this type of research design has only been used to investigate the role of prefrontal abnormalities in social cognitive dysfunction in schizophrenia. It was found^27^ that patients with schizophrenia and patients with ventromedial prefrontal cortical lesions showed similar impairments in affective, but not cognitive, mentalising, which suggests that affective mind-reading deficits may be a marker of fronto-limbic dysfunction in schizophrenia. Another study comparing patients with schizophrenia and patients with frontal lobe damage on a battery of emotion recognition tasks found that the performance of both groups was only partially consistent, suggesting that the social cognitive deficits observed in schizophrenia are not fully accounted for by frontal pathology^28^.

A number of studies have linked deficient mentalising in schizophrenia to abnormal patterns of activity in prefrontal and temporo-parietal regions^29^, but there has been little discussion of the amygdala and other subcortical structures in relation to mentalising in patients^30^. However, emotion recognition deficits in patients with schizophrenia have been associated with abnormal patterns of subcortical activity^24^. It can therefore be hypothesised that patients with schizophrenia will show deficits in inferring state of mind from the eyes, and these deficits are similar to those of patients with conditions linked to the direct disruption of medial temporal lobe activity, since the RMET task can be viewed as an interface between emotion processing and mentalising. In the research reported here, we examined this hypothesis by comparing the RMET performance of patients with schizophrenia with that of patients with MTLE and healthy controls.

It has been pointed out that the ToM computations may, in fact, tap distinctive, albeit partially overlapping, skills and that decomposition of the ToM construct into more-basic components associated with, for example, social perceptual, attentional and amnestic processes^31^, may be necessary to advance social neuroscience research. To gain insight into factors influencing mind reading capacity, we also examined the relationship between symptom severity in patients with schizophrenia and RMET performance and two aspects of cognitive functioning that are crucial to mind-reading, namely, psychomotor speed^32^ and executive functioning^33^.

## Results

### Statistical analysis

One-way ANOVA was used to investigate group differences in RMET scores. To maximise the reliability of the results, the Scheffé test was used for post hoc comparisons. The a priori planned comparisons between the HC, MTLE and SZ were post hoc expanded secondary comparisons accounting for the symptom profiles in patients with schizophrenia. Spearman rho’s coefficient was used in the correlation analysis of RMET scores and cognitive and clinical variables due to the non-normality of the distribution of the TMT-B scores and the PANSS subscales (Kolmogorov-Smirnov test, *p* < 0.05).

### Behavioural performance

**Figure 1:**
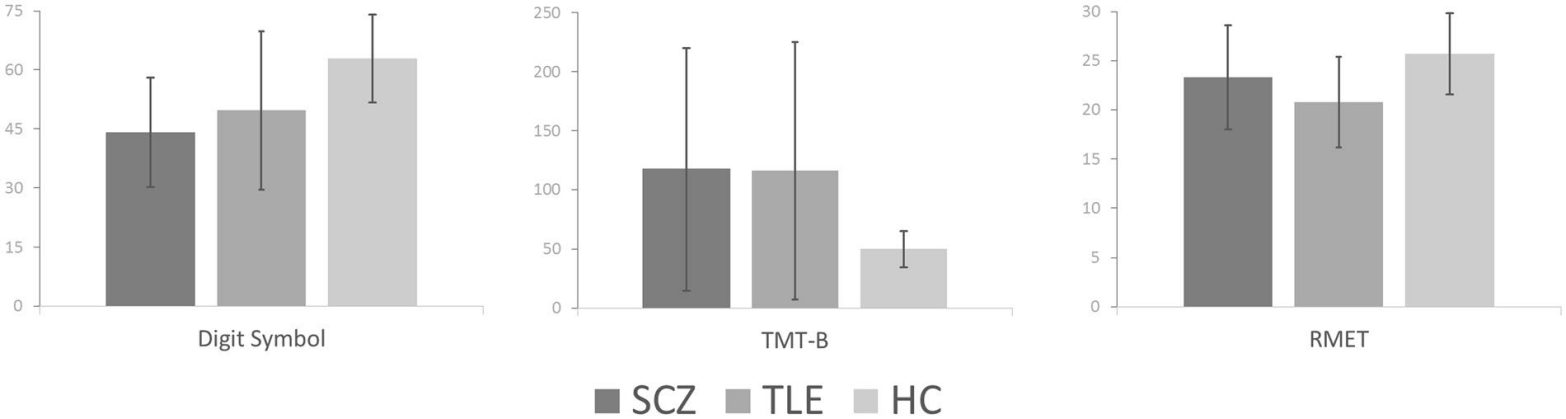
Results of cognitive (number of items coded for Digit Symbol; time in seconds for TMT-B) and mind-reading (number of items classified correctly in RMET) assessment. Error bars are for standard deviations. SCZ – patients with schizophrenia; MTLE – patients with temporal lobe epilepsy; HC – healthy controls.

A series of one-way ANOVAs comparing cognitive functions of the healthy and clinical groups showed that HCs performed better than both patient groups on the Digit Symbol test (*F*(2,125) = 20.0, p < 0.001) and TMT-B (*F*(2,125) = 9.4, *p* < 0.001). Moreover, one-way ANOVA comparing mind-reading abilities revealed a similar pattern of group differences on the RMET (*F*(2,125) = 10.2, *p* < 0.001). Post hoc tests confirmed that HCs had higher RMET scores than both patients with schizophrenia (*p* < 0.05) and patients with MTLE (*p* < 0.001), whilst the performance of the two clinical groups was similar (*p* > 0.05). All the results are depicted in Fig. 1.

### Correlation between tasks

Scores on the RMET and cognitive tasks (Digit Symbol, Trail Making Test -B) were not correlated in patients with MTLE or HCs. In patients with schizophrenia, the RMET score was associated with the score on the Digit Symbol test (rho = 0.42, *p* < 0.001) and the TMT-B performance time (rho = −0.32, *p* = 0.025).

### Relationship with symptoms

It has been postulated that both the negative and positive symptoms of schizophrenia are relevant to patients’ mind-reading performance^34^. In line with this, positive and negative symptoms have been shown to be associated with opposite patterns of mentalising problems^35^. Thus, we examined whether the subgroups of patients selected due to the symptom severity in this study would differ in RMET scores. Median-split was used with a cut-off score of 14 in the PANSS Positive subscale, thus indicating mild positive symptoms or lack of them in the low positive symptoms (POS-LO) group and moderate to severe positive symptoms in the high positive symptoms (POS-HI) group.

There were no differences in age and gender between the POS-LO and POS-HI groups and the other two groups; however, both the HCs and the POS-LO group were better educated than the POS-HI group (*F*(2,122) = 18.2, *p* < 0.001). Detailed demographic and clinical information on the POS-LO and POS-HI subgroups can be found in Table 1.

**Table 1:** Basic sociodemographic and clinical characteristics of the subgroups of patients with schizophrenia.

	SCZ POS_HI (n = 24)	SCZ POS_LO (n = 24)	
Age (years)	35.5 [10.5]	36.0 [6.4]	F(3,122) = 2.3 n.s.
Gender	12/12 (50%)	14/10 (58%)	Χ^2^ = 1.0 n.s.
Education	12.2 [1.9]	14.5 [2.6]	F(3,122) = 18.2***HC >SCZ POS_HI; MTLE; SCZ POS_LO >SCZ POS_HI
PANSS_POS	21.0 [3.8]	9.9 [1.8]	t(46) = 13.0***
PANSS_NEG	24.4 [8.8]	18.3 [4.3]	t(46) = 3.1**
PANSS_TOT	85.3 [19.1]	55.2 [8.7]	t(46) = 5.8***
Treatment			Χ^2^ = 0.6 n.s.
Monotherapy with SGA	12	13	
Polytherapy with SGA	11	8	
No medication	0	1	
Mono- or polytherapy with FGA	1	2	

All of the values are given in an M [SD] format with an exception for gender, where the M/F ratio is given. SCZ POS_HI – patients with a high level of positive symptoms; SCZ POS_LO – patients with a low level of positive symptoms.

Analysis of the RMET scores (F(3,122) = 10.4 p < 0.001) revealed no differences between HCs (RMET: 25.7 ± 4.1) and the POS-LO (RMET: 25.3 ± 4.8) group, and both groups outperformed the POS-HI (RMET: 21.3 ± 5.2) and MTLE (20.8 ± 4.6) groups.

To investigate whether the POS-LO vs. POS-HI differences in RMET performance can be attributed simply to a difference in clinical state, we carried out an analogous median-split based on PANSS Negative symptoms. Subsequent analyses revealed no difference in RMET performance between NEG-LO (RMET: 23.6 ± 4.4) and NEG-HI (RMET: 23.0 ± 6.2) groups (*t*(46) = 0.3, n.s.) and no differences between either the NEG subgroup or the MTLE and HC groups. There were also no group differences in RMET performance when patients with schizophrenia were divided according to their total PANSS score (PANSS-HI RMET: 22.2 ± 5.6 vs. PANSS-LO: 24.3 ± 4.9 *t*(46) = 1.4, n.s.).

## Discussion

This study tested the hypothesis that patients with schizophrenia have similar deficits in mind reading to those found in patients with other neuropsychiatric conditions associated with dysfunction of medial temporal lobe structures. In line with our hypothesis, both patients with MTLE and patients with schizophrenia showed impaired performance on the RMET. Further investigation of the latter group revealed that patients with a high level of psychotic symptoms exhibited similar deficits to patients with MTLE, whilst patients with few psychotic symptoms did not differ from healthy controls.

A meta-analysis of eight studies encompassing a total of 229 patients with schizophrenia and 191 controls found large effect sizes for the RMET task (*d* = 0.90)^25^. A recent meta-analysis of social cognitive skills in patients with temporal lobe epilepsy also reported that TLE has large effects on emotion processing (*d* = 0.79) and ToM functioning (*d* = 0.89)^36^. In line with these results, we found that patients with schizophrenia were impaired on the RMET compared with healthy controls, but the magnitude of this effect (*d* = 0.51) was less than half that for patients with MTLE (*d* = 1.12).

Previous studies comparing patients with schizophrenia and other clinical populations have produced similar results. Several papers have contrasted the RMET performance of patients diagnosed with schizophrenia with that of patients diagnosed with ASD and found similar impairments in mind-reading in both groups^37, 38^. Others^39^ compared the RMET performance of patients with first-episode schizophrenia, a psychiatric control group (patients with non-psychotic depression) and two healthy comparison groups (university undergraduates and community controls). Both healthy comparison groups outperformed patients with schizophrenia, but the two clinical groups showed similar mind-reading abilities. Another study reported that patients with schizophrenia performed worse on the RMET than patients with bipolar disorder (with or without psychotic features) who did not differ from healthy controls^40^.

To the best of our knowledge, no previous study has directly compared RMET performance in patients with schizophrenia and patients from neurological populations. In one study^27^, researchers found similar impairment profiles in patients with schizophrenia and patients with ventromedial prefrontal lesions, but not dorsolateral prefrontal or non-frontal lesions, on an affective, mentalising, eye gaze-based task. The results of our study imply that patients with schizophrenia and patients with medial temporal lobe pathology have similar mind-reading abilities and suggest that impaired RMET performance should be seen as a non-specific indicator of fronto-limbic pathology in neuropsychiatric conditions rather than a disease-specific impairment.

Importantly, the RMET score was associated with cognitive functioning in patients with schizophrenia, but no such relationship was observed in patients with MTLE and healthy controls. A long-standing debate about the nature of the relationship between social and non-social cognition has produced numerous arguments for treating social cognitive deficits as independent from general cognitive impairments in schizophrenia^4^. At the same time, research on patients with schizophrenia has consistently found a relationship between ToM abilities and general cognitive impairments^33^. When it comes to RMET specifically, mind-reading ability was previously linked to working memory^41^, psychomotor speed^41^ and executive functions^14, 41^ in patients with schizophrenia. Probably the strongest evidence that cognitive deficits are an important factor in RMET performance in schizophrenia comes from a three-year study of neuropsychological functioning in a cohort of patients with first-episode schizophrenia (*n* = 160) and matched healthy controls (*n* = 159). At all three time-points (baseline, 1- and 3-year follow-ups), patients displayed impaired mind-reading. This impairment was correlated with deficits in seven cognitive domains (verbal and visual learning; working memory; processing speed; executive functions; motor dexterity; attention), and processing speed was the sole cognitive predictor of mind-reading performance^32^. We have extended these results by showing that there is an association between cognitive functioning and mind-reading in patients with schizophrenia but not in another clinical population, with a similar profile of cognitive and social cognitive impairments.

Furthermore, in this study, we observed that when patients were classified according to their level of positive symptoms, there was a group difference in RMET performance, but no such difference emerged when patients were grouped according to their negative symptoms or overall psychopathology. Whilst some previous studies found no associations between RMET performance and any group of schizophrenia symptoms^41, 42^, negative correlations between positive symptoms and RMET performance have been reported in patients with schizophrenia^37^, young people seeking help for mental health problems^43^ and participants from the general population who displayed sub-clinical schizotypal traits^44^. Interestingly, in a group of adult patients with schizophrenia, there was a relationship between PANSS positive symptoms and eyes-based basic emotion recognition but not complex state attribution^45^. In another study^46^, no difference was observed between the RMET performance of groups of patients with non-deficit and deficit schizophrenia, with both patient groups being outperformed by healthy controls. Another study by the same research group found an association between negative symptom severity and RMET scores in patients with schizophrenia, both during the acute phase and during remission, but no such relationship for positive symptoms^47^. Moreover, whilst we found that patients with schizophrenia and more-pronounced positive symptoms showed an RMET performance similar to that of patients with MTLE, others^48^ found that the pattern of mind-reading impairments observed in high-functioning patients with ASD was more similar to that of schizophrenia patients with negative symptoms than that of patients with paranoia symptoms.

These discrepancies between literature findings can be attributed to the fact that the different symptom profiles have previously been linked to different mentalising impairments, with positive symptoms being linked to excessive attribution of mental states (“overmentalising”) and negative symptoms to deficient attribution of mental states (“undermentalising”)^34, 35^. Unfortunately, the construction of the RMET does not allow one to differentiate between over- and undermentalising. Further research in which participants are assessed using the RMET in combination with other ToM tasks would allow such differentiation.

It is important to note that when patients were grouped according to their level of positive symptoms, the POS-HI group also displayed higher levels of negative symptoms and general psychopathology than the POS-LO group. On this basis, one might hypothesise that the difference between these groups is largely a reflection of current clinical status rather an enduring difference in symptom profile. This raises a wider problem - whether the mind-reading deficits observed in patients with schizophrenia are a “state” or “trait” feature of the disorder. Patients with MTLE show mentalising deficits irrespective of whether they have received temporal lobectomy^36^, but a meta-analysis of research on mentalising and schizophrenia^25^ found that when the analysis is limited to studies of remitted patients with schizophrenia, the RMET deficit is smaller (*d* = 0.72) than in general studies of patients with schizophrenia (*d* = 0.90). Similarly, a longitudinal study reported that patients performed better on the RMET when clinically stable than when in the acute phase of the disorder. Still, in both assessments, patients performed worse than the controls^47^. However, other studies have failed to detect differences between the RMET performance of outpatients and inpatients with schizophrenia^49^ or longitudinal differences in a large cohort of first-episode patients followed over three years^32^. Moreover, modest mind-reading deficits have been observed in the healthy relatives of patients with schizophrenia^14, 50^ but not in clinically high-risk groups^51^. In summary, no conclusion can be drawn about whether mind-reading impairment represents a state or trait feature of schizophrenia. In this study, we observed greater impairment in the subgroup of patients manifesting a higher level of positive symptoms, whilst no such difference was detectable when patients were grouped according to level of negative symptoms or overall psychopathology. This pattern of results indicates that in our sample, the deficit in mind-reading cannot simply be attributed to the patient’s current clinical state.

Although this study examined well-powered clinical samples and used well-established measures to assess cognitive and mind-reading skills, it does have some limitations. Firstly, due to the brevity of the assessment, we were unable to include a more complex assessment of cognitive and social cognitive skills; thus, it was impossible to examine the relationship between RMET performance and performance on basic emotion recognition tasks (e.g., Facial Emotion Identification task)^14, 52, 53^ or complex ToM tasks (e.g., Frith-Happé animations)^14^. Moreover, we recruited both outpatients and inpatients with schizophrenia, thus increasing the heterogeneity of the schizophrenia group. Furthermore, we assessed symptoms of schizophrenia with the PANSS, which does not differentiate between reality distortion and disorganisation symptoms, and it has been shown that these two classes of symptom contribute differentially to the mentalising impairment of patients with schizophrenia^54^. Also important is that there were some within-group differences (e.g., types and number of seizures, types and doses of medication, shape and extent of the epileptic focus) due to the large diversity of epilepsy as a disease. This heterogeneity could also affect the performance on psychological tests, so it needs to be better controlled in future research.

The main observation of this study is the lack of difference between the mind-reading performance of patients with schizophrenia and patients with MTLE. Both disorders are characterised by marked limbic dysfunction, but in neither case are the alternations in neural activity exclusive to this brain system. The RMET task taps both higher-order mentalising abilities and lower-level facial emotion recognition processes, so impaired performance may be due to dysfunction of the amygdalar network, which is a feature of both diseases. Interestingly, a relationship between general cognitive impairments and mind-reading abilities was found only in patients with schizophrenia. In MTLE, it is usually possible to determine the main source of abnormal neural activity. It should also be noted that interaction between cognitive and socio-emotional processing in patients with schizophrenia has been linked to abnormal patterns of connectivity amongst the numerous neural systems involved in these functional domains. These include both cortical and subcortical structures with patterns of prefrontal-subcortical coupling considered as playing an important role in the disruption of interactions between emotional and cognitive processes in patients with schizophrenia^23, 55^. Importantly, we also found worse mind reading abilities in patients with more-pronounced positive symptoms compared to the group with milder positive symptoms. This finding can be seen as additional evidence that abnormal subcortical activity contributes to mind-reading deficits in schizophrenia. Although we cannot rule out the possibility that this effect is just a reflection of the generally worse clinical state of the group displaying more psychotic symptoms^47^, we consider these results strong evidence for the hypothesis that positive symptoms are linked to overmentalising. Although the mind-reading impairment in schizophrenia has both “state” and “trait” features, the results of this study suggest that RMET problems in patients with schizophrenia reflect a specific cluster of symptoms rather than a general clinical state.

In sum, our results suggest that although, considered as a single group, patients with schizophrenia display a stable mind-reading deficit that is comparable with that observed in patients with clearly defined medial temporal lobe pathology, there are a number of factors (e.g., poor general cognitive abilities, positive symptoms) that may be associated with more-severe impairments in mentalising in this group. We believe that the results of our study show that on the one hand, social cognitive deficits may be a ubiquitous problem across neuropsychiatric populations, while on the other hand, they may be differentially affected by various disorder-related factors in various clinical populations. Furthermore, the finding that RMET deficits can be found in patients with MTLE that cannot be clearly linked to the pathology in regions usually linked with ToM (mPFC, TPJ) suggests that targeting only specific cortical regions with brain stimulation methods^56^ may not be sufficient to cope with social cognitive deficits in patients with neuropsychiatric disorders. Thus, it may be suggested that only a combination of interventions that concurrently target social cognition^7^ and cognitive deficits (e.g., cognitive remediation^57^) and supplement them with methods that may be effective in reducing symptoms^56^ will be an effective way of coping with social cognitive deficits in patients.

## Methods

### Procedure and participants

All participants underwent a standardised interview and neuropsychological assessment, which took approximately thirty minutes. They were examined individually. Cognitive testing was performed immediately after the interview. First, the participants completed the Trail Making Test part B (TMT-B) and Digit Symbol subtests from WAIS-R in a paper-and-pencil format. Then, the Reading Mind in Eyes Test (RMET) was administered by an investigator.

Moreover, patients with schizophrenia were assessed with the Positive And Negative Syndrome Scale (PANSS)^58^. All procedures were carried out in accordance with the Declaration of Helsinki and were approved by the Ethics Committee of the Department of Psychology, University of Warsaw. All participants gave written, informed consent to participation.

### Patients with schizophrenia

Forty-eight inpatients (n = 26) or outpatients (n = 22) with paranoid schizophrenia who were clinically stable to an extent that allowed participation in the neuropsychological assessment were recruited from the Department of Old Age Psychiatry and Psychotic Disorders, Medical University of Lodz, and the Third Department of Psychiatry, Institute of Psychiatry and Neurology in Warsaw. The group consisted of twenty-six men and twenty-two women aged twenty-three to fifty-eight years, with eight to seventeen years of formal education. To be eligible to participate in the study, patients had to be aged between eighteen and fifty-five years and had to be diagnosed during a clinical interview by trained psychiatrists (AW and MJ) as having chronic paranoid schizophrenia according to ICD-10 criteria. These criteria include the presence of at least one of the following symptoms: a) thought insertion/withdrawal/broadcasting; b) delusions of control, clearly indicated by the patient’s body and thoughts, as well as delusional perception; c) hallucinatory voices; or d) other persistent socially inappropriate delusions; or they had to have at least two of the following symptoms: a) persistent hallucinations in any modality without clear affective content or accompanied by persistent over-valued ideas; b) neologisms in speech; c) catatonic behaviour; or d) negative symptoms (e.g., apathy, scant speech and emotional responses)^59^.

The exclusion criteria were a history of other neurological or psychiatric disorders, treatment with electroconvulsive therapy or drug or alcohol abuse. All but one of the participants were receiving antipsychotic treatment at the time of the study: twenty-five patients were receiving second-generation antipsychotics (SGAs) as monotherapy, nineteen were taking two or more SGAs and three were treated with a combination of SGA and typical antipsychotics.

### Mesial temporal lobe epilepsy patients

We also recruited thirty-one patients with drug-resistant MTLE who had been admitted to the Department of Neurosurgery at the Medical University of Warsaw for pre-surgical evaluation. This patient group consisted of seventeen women and fourteen men aged eighteen to fifty-one years, with eight to seventeen years of formal education. The average age at onset of epilepsy was twelve years (range: 3 months -31 years), and the average number of seizures per month was twenty-three (range: 2–200). According to the International League Against Epilepsy’s revised operational classification of seizures^60^, all patients had focal seizures with impaired awareness (complex partial), twenty-eight patients also had focal aware seizures (aura), and nine patients had focal seizures generalised to bilateral tonic-clonic seizures. Patients were treated with monotherapy (*n* = 2) or polytherapy (two drugs: *n* = 16; three drugs: *n* = 13). Sixteen patients had left*-*sided epileptic foci, nine had right-sided foci, and six had bilateral foci. The exclusion criteria were the same as for the group of patients with schizophrenia.

### Healthy controls

Healthy participants (n = 47; 25 men 22 women) were volunteers with no known history of psychiatric or neurological disorders recruited from the community via online advertisements. They were screened for the presence of psychiatric symptomatology by the investigator. We did not use any particular questionnaire or scale during the clinical interview. The age of healthy controls (HC) was twenty-one to sixty years, and they had twelve to seventeen years of formal education. All participants had normal or corrected-to-normal vision.

### Demographic data analysis

A series of one-way ANOVAs comparing the age and gender distribution of the HCs (32.3 ± 9.1 yrs; 25 M, 22 F) with the patients with schizophrenia (35.8 ± 8.6 yrs; 26 M, 22 F) or MTLE (30.9 ± 7.7; 14 M, 17 F) revealed that the groups did not differ significantly.

However, ANOVAs comparing the age and gender distribution of the MTLE and schizophrenia groups showed that patients with MTLE were younger than patients with schizophrenia (*F*(2,125) = 3.5, *p* < 0.05).

Moreover, as shown by one-way ANOVA, both patients with schizophrenia (edu: 13.4 ± 2.5 yrs.) and patients with MTLE (edu: 13.0 ± 3.0 yrs.) had fewer years of education than healthy controls (edu: 15.9 ± 1.8; *F*(2,125) = 19.3, *p* < 0.001). Detailed demographic information on the groups can be found in Table 2.

**Table 2:** Basic sociodemographic and clinical characteristics of the participants of the study.

	SCZ (n = 48)	MTLE (n = 31)	HC (n = 47)	
Age (years)	35.8 [8.6]	30.9 [7.7]	32.3 [9.1]	F(2,125) = 2.3 n.s.
Gender	26/22 (54%)	14/17 (45%)	25/22 (53%)	Χ^2^ = 0.6 n.s.
Education	13.4 [2.5]	13.0 [2.9]	15.9 [1.8]	F(2,125) = 19.3*** HC > SCZ, MTLE

All of the values are given in M [SD] format with the exception of gender, where the M/F ratio is given. SCZ – patients with schizophrenia; MTLE – patients with mesial temporal lobe epilepsy; HC – healthy controls.

### Psychological assessment

#### Mentalising

The Reading Mind in Eyes Test, which is based on the ToM concept, is a diagnostic tool used to evaluate the accuracy of an individual’s attributions of emotions and mental states to other people (mentalising/mind reading). The stimuli are thirty-six black-and-white photographs of the region around the eyes (19 male and 17 female cases) surrounded by four words describing various mental states (e.g., serious; ashamed; alarmed; bewildered). The subject is required to choose the word that best describes the person in each picture^8^. Because the RMET is a forced-choice test with four alternatives, the chance level of accuracy is 25%.

#### Cognitive assessment

Psychomotor speed was assessed with the Digit Symbol test, a subtest of the Wechsler Adult Intelligence Scale-Revised. The stimuli are ninety-three randomly chosen digits ranging from 1 to 9. Each digit has a matching symbol, and the subject is required to write the appropriate symbol under each digit. There is a time limit of ninety seconds, and the subject has to work as fast as possible^61^.

Executive functioning was assessed with the Trail-Making Test part B (TMT-B). The stimuli for the TMT-B are twenty-five circles each containing a single letter (A-L) or number (1–13) distributed across a sheet of paper. The subject has to link the circles in the correct order, alternating between letters and numbers (1-A-2-B… etc.)^61^.

#### Assessment of psychopathological symptoms

The Positive And Negative Syndrome Scale (PANSS) consists of three subscales: Positive and Negative (both 7 items) and General Psychopathology (16 items). It is used to assess symptom severity in patients with schizophrenia and takes the form of a clinical interview conducted by a trained specialist. The subject is given a score of between 1 and 7 on each item; higher scores represent higher levels of symptoms^58^. The PANSS Positive symptoms scale, which was used for the median-split during this study, consists of seven items associated with a wide range of psychotic and excitability symptoms (delusions, conceptual disorganisation, hallucinations, excitement, grandiosity, suspiciousness and hostility).
